# Effect of fluid administration on scene to traffic accident patients by EMS personnel: a propensity score-matched study using population-based ambulance records and nationwide trauma registry in Japan

**DOI:** 10.1007/s00068-020-01590-z

**Published:** 2021-01-25

**Authors:** Yusuke Katayama, Tetsuhisa Kitamura, Kosuke Kiyohara, Kenichiro Ishida, Tomoya Hirose, Shunichiro Nakao, Jotaro Tachino, Tasuku Matsuyama, Takeyuki Kiguchi, Yutaka Umemura, Tomohiro Noda, Yuko Nakagawa, Takeshi Shimazu

**Affiliations:** 1 Department of Traumatology and Acute Critical Medicine, Osaka University Graduate School of Medicine, 2-15, Yamada-oka, Suita, 565-0871 Japan; 2 Division of Environmental Medicine and Population Sciences, Department of Social and Environmental Medicine, Osaka University Graduate School of Medicine, 2-15, Yamada-oka, Suita, Japan; 3 Department of Food Science, Faculty of Home Economics, Otsuma Women’s University, 12, Sanban-cho, Chiyoda-ku, Tokyo, Japan; 4 Department of Acute Medicine and Critical Care Medical Center, Osaka National Hospital, National Hospital Organization, 2-1-14, Hoenzaka, Chuo-ku, Osaka, Japan; 5 Department of Emergency Medicine, Kyoto Prefectural University of Medicine, 465 Kajiicho, Hiroko-ji Noboru, Kawaramachi-dori, Kamigyo-ku, Kyoto, Japan; 6 Division of Trauma and Surgical Critical Care, Osaka General Medical Center, 3-1-56, Mandai-higashi, Sumiyoshi-ku, Osaka, Japan; 7 Kyoto University Health Services, Yoshida-honmachi, Sakyo-ku, Kyoto, Japan; 8 Department of Traumatology and Critical Care Medicine, Osaka City University Graduate School of Medicine, 1-5-7, Asahi-machi, Abeno-ku, Osaka, Japan

**Keywords:** Emergency medical service, Trauma, Prehospital fluid administration, Paramedic, Shock

## Abstract

**Purpose:**

The aim of this study was to assess the effect of fluid administration by emergency life-saving technicians (ELST) on the prognosis of traffic accident patients by using a propensity score (PS)-matching method.

**Methods:**

The study included traffic accident patients registered in the JTDB database from January 2016 to December 2017. The main outcome was hospital mortality, and the secondary outcome was cardiopulmonary arrest on hospital arrival (CPAOA). To reduce potential confounding effects in the comparisons between two groups, we estimated a propensity score (PS) by fitting a logistic regression model that was adjusted for 17 variables before the implementation of fluid administration by ELST at the scene.

**Results:**

During the study period, 10,908 traffic accident patients were registered in the JTDB database, and we included 3502 patients in this study. Of these patients, 142 were administered fluid by ELST and 3360 were not administered fluid by ELST. After PS matching, 141 patients were selected from each group. In the PS-matched model, fluid administration by ELST at the scene was not associated with discharge to death (crude OR: 0.859 [95% CI, 0.500–1.475]; *p* = 0.582). However, the fluid group showed statistically better outcome for CPAOA than the no fluid group in the multiple logistic regression model (adjusted OR: 0.231 [95% CI, 0.055–0.967]; *p* = 0.045).

**Conclusion:**

In this study, fluid administration to traffic accident patients by ELST was associated not with hospital mortality but with a lower proportion of CPAOA.

## Introduction

Death due to hemorrhage is an important issue in trauma care around the world. Indeed, it was reported that approximately 40% of trauma deaths occurring within 24 h were due to hemorrhage [1]. In the 1970s in the United States, fluid resuscitation prior to hospital arrival was considered an important component of prehospital care for trauma patients. In the Advanced Trauma Life Support guidelines from the American College of Surgeons, initial resuscitation with crystalloid fluid still begins with a 1-L bolus of warmed isotonic fluid [2]. Maintaining systolic blood pressure to stabilize tissue circulation and prevent acidosis is associated with a favorable prognosis for trauma patients. However, several observational studies and clinical trials have reported that aggressive fluid administration prior to definite care may actually increase mortality in trauma patients [3–5]. Aggressive fluid administration to maintain blood pressure may contribute to bleeding due to pro-inflammatory effects and coagulopathy caused by hemodilution and hypothermia. In addition, several studies reported that patient transport from the scene to the hospital is delayed when paramedics secure intravenous lines in a prehospital setting [6–8].

In Japan, the regulations for emergency life-saving technicians (ELST) were amended in 2014 to allow ELST to secure intravenous lines and administer lactated Ringer’s solution to patients in shock or with crush syndrome [9]. However, it is unknown whether fluid administration by ELST in Japan has contributed to the improvement of outcomes in trauma patients.

The emergency medical service (EMS) in Japan is a public service provided by the local government, and all ambulance records are reported to the Fire and Disaster Management Agency (FDMA). The Japanese Trauma Data Bank (JTDB) is the nationwide trauma registry in Japan, and about 300,000 trauma patients were registered by 2017. The aim of this study was to assess the effect of fluid administration by ELST on the prognosis of traffic accident patients by linking population-based ambulance records and the nationwide trauma registry in Japan based on a propensity score (PS)-matching method.

## Materials and methods

### Study design, population and setting

This was a retrospective observational study using ambulance records in Japan and data registered in the JTDB database. The study period was 2 years from January 2016 to December 2017. We included traffic accident patients among the trauma patients registered in the JTDB. We excluded patients with an Injury Severity Score (ISS) of < 16, patients in cardiopulmonary arrest (CPA) at the time of ambulance arrival at the scene, and patients with insufficient data on date, vital signs at hospital arrival, and prognosis in this study. Patients in CPA at the time of ambulance arrival at the scene were defined as patients for whom the EMS personnel at the scene provided pre-hospital care for CPA, such as chest compressions, airway clearance, and administration of adrenaline. This study was approved by the ethics committee of Osaka University Graduate School of Medicine (approval no. 20233). Because the JTDB data and ambulance records that we were provided from the FDMA were anonymized, the requirement of informed consent was waived. This article was written based on the STROBE statement to assess the reporting of cohort and cross-sectional studies [10].

### Emergency medical service and ambulance records in Japan

The EMS system and ambulance record in Japan were previously described in detail [11]. The EMS system in Japan is operated by local fire departments and is activated by a 1-1-9 call from anywhere in Japan [12]. In 2016, there were 733 fire department headquarters and 1714 fire stations with 6210 ambulances throughout Japan [13]. Life support is provided 24 h a day. Usually, each ambulance has a crew of three emergency providers including at least one ELST, a highly trained prehospital emergency care provider [14]. In Japan, most emergency providers who have worked in the fire department for a certain period of time attend lectures on emergency medical care at a specialized facility and then go on to receive practical training on emergency care such as intubation and IV access at an emergency medical facility to qualify for the national examination. Those who pass the national examination can become an ELST.

Ambulance records are collected annually for statistical and administrative purposes in all prefectures of Japan via a standardized electronic form. EMS personnel submit the ambulance record to the local fire station. All emergency patients who required EMS for transport by ambulance to a particular institution were captured. Then, all ambulance records were collected in the FDMA under the Ministry of Internal Affairs and Communications in Japan. The data used in this study were provided from the FDMA after all personal identifiers were removed.

### Japanese trauma data bank

The JTDB was established in 2003 by the Japanese Association for the Surgery and Trauma (Trauma Surgery Committee) and the Japanese Association for Acute Medicine (Committee for Clinical Care Evaluation) [15, 16] and is similar to trauma databases in North America, Europe, and Oceania [17]. By 2016, JTDB data had been registered by 256 major emergency medical institutions around Japan [18]. These hospitals have ability equal to Level I trauma centers in the United States. Data were collected via the Internet from participating institutions. In most cases, the physicians and technician who attended an AIS coding course registered the data [17].

The JTDB captures data from trauma patients that includes age, sex, type of patient, Abbreviated Injury Score (AIS code; version 1998), ISS, vital signs on hospital arrival, date and time course from hospital arrival to discharge, medical treatments such as interventional radiology and CT scanning, and complications in accordance with regular forms with coding items [19]. The ISS was calculated from the top three scores of the AIS for nine sites classified by AIS codes.

### Patient selection

We selected patients from ambulance records in Japan for whom a traffic accident was the reason for the ambulance call. Next, we selected patients from the JTDB whose type of patient was car driver, front-seat passenger, back seat passenger, motorcycle rider, pillion passenger, bicyclist, pedestrian, or other vehicle passenger. Then, all identified patients were matched automatically and manually confirmed using patient data such as date and time of hospital arrival and identifying patient information such as age and sex. Age differences of up to 2 years were allowed. All data that did not match between the two datasets were excluded from this study.

### Endpoint

The main outcome was hospital mortality, which was defined from data recorded in the JTDB database. The secondary outcome was CPA on hospital arrival (CPAOA), which was defined as a patient systolic blood pressure of 0 mmHg or heart rate of 0 bpm on hospital arrival.

### Statistical analysis

Prehospital characteristics and outcome were evaluated between the group of patients with fluid administration by ELST (fluid group) and the group of patients without fluid administration by ELST (no fluid group). Multivariable logistic regression analysis was used to assess the impact of fluid administration by ELST on the prognosis of trauma patients; odds ratios and their 95% confidence intervals were calculated.

Potential confounding factors that were adjusted for in the multivariable analyses included age (continuous variable), sex (male or female), time of day (daytime or nighttime), weekend (weekend or weekday), shock at the scene (presence or absence), ungaugeable blood pressure or missing (presence or absence), type of patient (car driver, front-seat passenger, back seat passenger, motorcycle rider, pillion passenger, bicyclist, pedestrian, and other vehicle passenger), ISS (continuous variable), presence of injury with an AIS of 3 or more (head, neck, face, thorax, abdomen, spine, upper extremity, and lower extremity including pelvis), and time interval from ambulance call to arrival at the scene (continuous variable). This study defined daytime as 09:00 to 17:59 and nighttime as 18:00 to 08:59 and defined shock as systolic blood pressure below 80 mmHg at the scene [20].

We estimated a PS by fitting a logistic regression model that adjusted for the 17 variables listed above. We also performed a receiver operating characteristic curve analysis with an area under the curve of the PS to predict the implementation of fluid administration by ELST among traffic accident patients. One-to-one pair matching between the fluid group and the no fluid group was performed by nearest-neighbor matching without replacement, with the use of a caliper width equal to 0.2 of the standard deviation of the logit of the PS. Covariate balances before and after matching were checked by comparison of standardized mean differences (SMD) [21]. A SMD of < 0.25 was considered to indicate a negligible imbalance between the two groups [22]. We investigated the association between the implementation of fluid administration by ELST and hospital mortality among traffic accident patients using univariate and multivariate logistic regression analyses. In addition, we performed a subgroup analysis by severe head injury (head AIS of 3 or more or not). All tests were two tailed, and *p* values of < 0.05 were considered statistically significant. All statistical analyses were performed with SPSS version 23.0J (IBM Corp., Armonk, NY, USA) and R version 3.1.0 (The R Foundation for Statistical Computing).

## Results

**Fig. 1:**
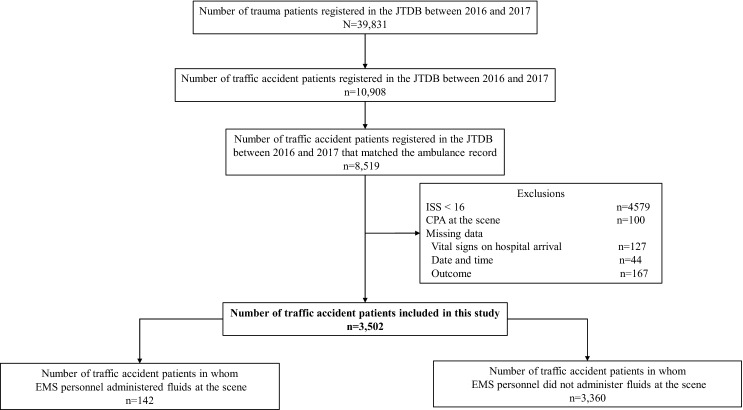
Flowchart of patient enrollment

Figure 1 shows the patient flow in this study. From January 2016 to December 2017, 39,831 trauma patients were registered in the JTDB database, of whom 10,908 were traffic accident patients. Of these patients, 8519 were matched with ambulance records provided from the FDMA. We excluded the patients with an ISS of < 16 (*n* = 4579), patients in CPA at the time of ambulance arrival at the scene (*n* = 100), and patients with insufficient data (vital signs at hospital arrival, *n* = 127; date, *n* = 44; prognosis, *n* = 167) and thus included 3502 patients in this study. Of these patients, 142 patients were given fluid administration by ELST and 3360 patients were not given fluid administration by ELST.

Table 1 shows the clinical characteristics of the patients before and after PS matching. A higher population of males and more nighttime accidents were found in the fluid group than in the no fluid group, and the ISS was higher in the fluid group than that in the no fluid group before PS matching. In addition, there was a high percentage of shock and ungaugeable blood pressure or missing data at the scene in the fluid group and a high percentage of car drivers and patients with thorax injury and abdominal injury. After PS matching, 141 patients were selected from each group, and the area under the curve in the logistic regression model for PS calculation was 0.776. The covariates were improved among the groups after PS matching.

**Table 1:** Patient characteristics among the full cohort and the propensity score-matched cohort

	All patients					Propensity score-matched patients				
	Infusion by EMS personnel (+)		Infusion by EMS personnel (−)		SMD	Infusion by EMS personnel (+)		Infusion by EMS personnel (−)		SMD
	(*N* = 142)		(*N* = 3360)			(*N* = 141)		(*N* = 141)		
Age, mean (SD)	52.1 (21.5)		53.6 (23.4)		0.067	52.1 (21.5)		50.8 (23.2)		0.058
Male, *n* (%)	102 (71.8)		2237 (66.6)		0.114	101 (71.6)		103 (73.0)		0.032
Weekend, *n* (%)	46 (32.4)		1007 (30.0)		0.052	46 (32.6)		46 (32.6)		0.091
Nighttime (18:00–7:59), *n* (%)	84 (59.2)		1674 (49.8)		0.188	83 (58.9)		81 (57.4)		0.029
Systolic blood pressure on the scene										
Shock (<80 mmHg) on scene	22 (15.5)		62 (1.8)		0.500	21 (14.9)		19 (13.5)		0.041
Ungaugeable or missing	41 (28.9)		499 (19.3)		0.344	41 (29.1)		39 (27.7)		0.031
Type of patient										
Car driver	56 (39.4)		648 (19.3)		0.454	55 (39.0)		53 (37.6)		0.029
Front seat passenger	5 (3.5)		128 (3.8)		0.015	5 (3.5)		5 (3.5)		0.000
Back seat passenger	3 (2.1)		108 (3.2)		0.068	3 (2.1)		2 (1.4)		0.054
Motorcycle rider	22 (15.5)		819 (24.4)		0.224	22 (15.6)		27 (19.1)		0.091
Pillion passenger	1 (0.7)		31 (0.9)		0.024	1 (0.7)		0 (0)		0.120
Bicyclist	17 (12.0)		690 (20.5)		0.234	17 (12.1)		18 (12.8)		0.022
Pedestrian	38 (26.8)		896 (26.7)		0.002	38 (27.0)		36 (25.5)		0.032
Other vehicle passenger	0 (0)		40 (1.2)		0.155	0 (0)		0 (0)		–
ISS, mean (SD)	31.1 (12.9)		26.5 (11.5)		0.376	31.2 (12.9)		32.3 (16.8)		0.073
Number of patients with AIS ≥3 by body region, *n* (%)										
Head	59 (41.5)		1915 (57.0)		0.313	59 (41.8)		57 (40.4)		0.029
Neck	3 (2.1)		56 (1.7)		0.033	3 (2.1)		3 (2.1)		0.000
Face	1 (0.7)		14 (0.4)		0.039	1 (0.7)		1 (0.7)		0.000
Thorax	101 (71.1)		1846 (54.9)		0.340	100 (70.9)		106 (75.2)		0.096
Abdomen	34 (23.9)		376 (11.2)		0.340	34 (24.1)		36 (25.5)		0.033
Spine	22 (15.5)		445 (13.2)		0.064	22 (15.6)		18 (12.8)		0.081
Upper extremity	8 (5.6)		143 (4.3)		0.064	8 (5.7)		11 (7.8)		0.085
Lower extremity including pelvis	53 (37.3)		740 (22.0)		0.340	52 (36.9)		54 (38.3)		0.029
Time interval from ambulance call to arrival on scene (min), mean (SD)	10.4 (6.7)		9.3 (5.1)		0.185	10.5 (6.7)		9.9 (5.7)		0.096

*EMS* emergency medical service, *SMD* standardized mean difference, *SD* standard deviation, *ISS* injury severity score, *AIS* abbreviated injury scale

Table 2 shows the relationship between fluid administration by ELST at the scene and discharge to death. In the univariate regression analysis of the total cohort, the fluid group had a higher rate of discharge to death than the no fluid group (23.2% [33/142] vs 15.2% [512/3360], crude OR: 1.684, *p* = 0.011). In the multivariate logistic regression analysis, fluid administration by ELST at the scene was not associated with discharge to death (adjusted OR: 1.088 [95% CI, 0.658–1.798]; *p* = 0.743). In the PS-matched model, the proportions of discharge to death in the fluid group and the no fluid group were similar (23.4% [33/141] vs 26.2% [37/141]), and there was no significant relationship between fluid administration by ELST at the scene and discharge to death (crude OR: 0.859 [95% CI, 0.500–1.475]; *p* = 0.582). Fluid administration by ELST at the scene was also not associated with discharge to death in the multivariate logistic regression model (adjusted OR: 0.980 [95% CI, 0.443–1.986]; *p* = 0.955).

**Table 2:** Survival outcomes of traffic accident patients with or without fluid administration by ELST

	Total	With prehospital infusion	Without prehospital infusion	Crude OR	(95% CI)	Adjusted OR	(95% CI)^a^
All patients^a^	(*N* = 3502)	(*N* = 142)	(*N* = 3360)				
Discharge to death	545 (15.6%)	33 (23.2%)	512 (15.2%)	1.684	(1.128–2.514)	1.088	(0.658–1.798)
Propensity score-matched patients^a^	(*N* = 282)	(*N* = 141)	(*N* = 141)				
Discharge to death	70 (25.5%)	33 (23.4%)	37 (26.2%)	0.859	(0.500–1.475)	0.980	(0.483–1.986)

ORs were calculated for traffic accident patients with versus without fluid administration by EMS personnel at the scene

*EMS* emergency medical service, *OR *odds ratio, *CI* confidence interval

^a^Adjusted for age, sex, time of the day, day of the week, ISS, AIS score by each body region, shock at the scene, mechanism of injury, and time interval from ambulance call to arrival at the scene

Table 3 shows the relationship between fluid administration by ELST at the scene and CPAOA. In the univariate regression analysis of the total cohort, there was little difference in the proportion of CPAOA in the fluid group compared with the no fluid group (5.6% [8/142] vs 4.0% [134/3360], crude OR: 1.134 [95% CI, 0.547–2.354]; *p* = 0.735). In the multivariate logistic regression model, the fluid group tended to have a better prognosis than the no fluid group, but the difference was not statistically significant (adjusted OR: 0.443 [95% CI, 0.185–1.060]; *p* = 0.067). However, in the PS-matched model, the fluid group had a smaller proportion of CPAOA than the no fluid group (5.7% [8/141] vs 12.1% [17/141], crude OR: 0.439 [95% CI, 0.183–1.053]; *p* = 0.064). The fluid group had a statistically better prognosis than the no fluid group in the multiple logistic regression model (adjusted OR: 0.231 [95% CI, 0.055–0.967]; *p* = 0.045).

**Table 3:** CPAOA of traffic accident patients with or without fluid administration by ELST

	Total	Fluid group	No fluid group	Crude OR	(95% CI)	Adjusted OR	(95% CI)^a^
All patients^a^	(*N* = 3502)	(*N* = 142)	(*N* = 3360)				
CPAOA	176 (5%)	8 (5.6%)	134 (4%)	1.134	(0.547–2.354)	0.443	(0.185–1.060)
Propensity score-matched patients^a^	(*N* = 282)	(*N* = 141)	(*N* = 141)				
CPAOA	25 (9.1%)	8 (5.7%)	17 (12.1%)	0.439	(0.183–1.053)	0.231	(0.055–0.967)

ORs were calculated for traffic accident patients with versus without fluid administration by EMS personnel at the scene

*EMS* emergency medical service, *OR* odds ratio, *CI* confidence interval, *CPAOA* cardiopulmonary arrest on hospital arrival

^a^Adjusted for age, sex, time of the day, day of the week, ISS, AIS score by each body region, shock at the scene, mechanism of injury, and time interval from ambulance call to arrival at the scene

Table 4 shows the results of subgroup analysis divided into patients with and without severe head injury. Fluid administration by ELST was not associated with discharge to death in either the patients with severe head injury (AOR: 1.081 [95%CI, 0.526–2.223], *p* = 0.832) or in those without severe head injury (AOR: 1.046 [95%CI, 0.481–2.271], *p* = 0.910). However, the proportion of CPAOA tended to be lower in the fluid group among patients without severe head injury, although this was not significant in the multivariate regression model (AOR: 0.231 [95% CI, 0.044–1.216], *p* = 0.084).

**Table 4:** Survival outcomes and CPAOA of traffic accident patients with severe head injury or not

	Total	With prehospital infusion	Without prehospital infusion	Crude OR	(95% CI)	Adjusted OR	(95% CI)^a^
Head injury over AIS score 3							
All patients^a^	(*N* = 1974)	(*N* = 55)	(*N* = 1919)				
Discharge to death	399 (20.2%)	15 (27.3%)	384 (20%)	1.499	(0.820–2.742)	1.081	(0.526–2.223)
CPAOA	109 (5.5%)	0 (0%)	109 (5.7%)	–	–	–	–
Head injury under AIS score 2							
All patients^a^	(*N* = 1528)	(*N* = 80)	(*N* = 1448)				
Discharge to death	146 (9.6%)	13 (16.3%)	133 (9.2%)	1.918	(1.032–3.567)	1.046	(0.481–2.271)
CPAOA	67 (4.4%)	2 (2.5%)	65 (4.5%)	0.546	(0.131–2.269)	0.231	(0.044–1.216)

ORs were calculated for traffic accident patients with versus without fluid administration by EMS personnel at the scene

*EMS* emergency medical service, *OR* odds ratio, *CI* confidence interval

^a^Adjusted for age, sex, time of the day, day of the week, ISS, AIS score by each body region, shock at the scene, mechanism of injury, and time interval from ambulance call to arrival at the scene

## Discussion

In this study, we found that fluid administration by ELST to traffic accident victims at the scene decreased the proportion of CPAOA but did not improve the mortality of these patients. In addition, both the time interval from ambulance call to hospital arrival and that between departure from the scene and hospital arrival were significantly longer in the group with fluid administration by ELST than those in the group without fluid administration by ELST. This study, which found that fluid administration by ELST improved the status of patients on hospital arrival, may be useful in examining the treatment for trauma patients by EMS personnel in the prehospital situation.

First, fluid administration by ELST was shown to be neither beneficial nor harmful to traffic accident patients in this study. In a subgroup analysis divided by severe head injury, fluid administration by ELST was neither beneficial nor harmful in either the patients with severe head injury or in those without severe head injury. There are several studies on intravenous access and fluid administration in prehospital settings. Some studies reported that fluid administration by EMS personnel to trauma patients had no effect on mortality [7, 23, 24], whereas other studies reported rather that it increased the mortality [6, 8]. Another report showed that the inability to obtain peripheral venous access is associated with the severity of injury [25]. As the present study included only traffic accident patients, all of the patients had suffered blunt trauma. This may be the reason why the present results differed from those of some of the previous studies.

Second, the proportion of CPAOA was lower in the group with fluid administration by ELST than that in the group without fluid administration in this study. In addition, fluid administration by ELST tended to be associated with a lower proportion of CPAOA among the patients without severe head injury. These results may be influenced by fluid administration by ELST and the maintenance of circulation in severe trauma patients. Seamon et al. reported that prehospital procedures were the only predictor of hospital mortality in a study of trauma patients undergoing emergency department thoracotomy, for which they compared ambulance versus non-ambulance transport methods such as a police car or private vehicle [6]. Although this previous study included trauma patients with emergency department thoracotomy, the present study included traffic accident patients, and not all of them underwent emergency department thoracotomy. Thus, it is possible that the urgency of the patients in the present study was less than that in the previous study, which may have led to a difference in these results. In a previous study by Sampalis et al., prehospital infusion provided no benefit when the prehospital time was < 30 min, and conversely, prehospital infusion increased the risk of mortality if prehospital time was longer than 30 min [8]. In that study, the mean ISS for patients with prehospital infusion was 15, and that for patients without prehospital infusion was 9. In contrast, among the PS-matched patients in the present study, the mean ISS was 31.3 for the patients in the fluid group and 30.3 for those in the no fluid group. Thus, because there were more severe trauma patients in this study than in the previous study, obtaining venous access in the prehospital setting may be useful for maintaining the circulatory system of severe trauma patients during their transport to the hospital. In addition, the prognosis of trauma patients is affected not only by prehospital care but also by surgical treatment and intensive care received in the hospital. Therefore, it may be difficult to assess the effect only of fluid administration by ELST or paramedics on hospital mortality. Indeed, other previous studies reported that prehospital time was longer in patients with versus without venous access obtained by paramedics and that mortality was same in the patients regardless of whether venous access was obtained by the paramedics [23, 24]. Therefore, in the present study, the proportion of patients with CPAOA was used as a measure to evaluate fluid administration by ELST because CPAOA is not affected by surgical treatment and intensive care. The results in the present study that fluid administration by ELST was associated with a lower proportion of CPAOA would suggest that fluid administration by ELST may contribute to the improved outcome of trauma patients. In addition, intraosseous infusion may improve the prognosis of trauma patients because it reduces the time interval to secure an infusion route. Furthermore, the method, dosage, and timing of administration of infusion products used in prehospital settings were evaluated in several studies [3, 7, 8, 23–25]. Although the method and dosage of infusion products are determined by local medical controls in Japan, it is mentioned that a bolus dose of 250–500 mL of fluid should be administered to trauma patients with a systolic blood pressure of < 80 mmHg in the Japan Prehospital Trauma Evaluation and Care (JPTEC) program that most emergency care providers attend [26]. These differences between the method and timing of fluid administration in the prehospital setting may have influenced the difference in results between the present study and these previous studies.

### Limitations

There are several limitations to this study. First, this study was limited to traffic accident patients to allow merging of ambulance records and trauma registry data. Therefore, although all of the traffic accident patients suffered blunt trauma, some of these patients may have also sustained penetrating trauma from car parts or a guardrail destroyed in the traffic accident. We also did not include patients with penetrating trauma such as gunshot or stab wounds. Therefore, we could not evaluate the effect of fluid administration in the prehospital setting on patients with penetrating trauma. Second, because ELST in Japan can only administer lactated Ringer’s solution, we could not evaluate the effect of the administration of other solutions such as normal saline. Third, it is unclear whether the cause of CPAOA was due to exsanguinating hemorrhage. In fact, about 40% of the patients included in this study suffered head trauma, so it is possible that this condition was the cause of CPAOA. Finally, this was an observational study, and therefore, we could not adjust for the effects of unknown confounding factors.

## Conclusion

In this study, we revealed that fluid administration by ELST to traffic accident patients at the scene was associated with a low proportion of CPAOA but was not associated with improved hospital mortality.
